# Driving Advice Documentation in Patient Notes and Discharge Summaries in Cardiology Patients

**DOI:** 10.7759/cureus.107148

**Published:** 2026-04-16

**Authors:** Kelvin Mupunga

**Affiliations:** 1 Medicine, Northern Ireland Medical and Dental Training Agency (NIMDTA), Belfast, GBR

**Keywords:** cardiac conditions, discharge summaries and patient notes, documentation, driver and vehicle licensing agency (dvla), driving restrictions

## Abstract

Background

Cardiac conditions can cause sudden symptoms that may impact driving, so the Driver and Vehicle Licensing Agency (DVLA) imposes restrictions on affected patients or those who have had cardiac procedures. Even though patients must inform DVLA, it is the doctors who are responsible for advising patients about these restrictions. Clinical practice experience and previously published reports show that driving advice was often missing from patient notes and discharge summaries. The quality improvement project (QIP) checked these records to see whether driving advice was documented.

Methods

A QIP using the Plan-Do-Study-Act (PDSA) cycle was conducted with the aim of assessing the documentation of driving advice in cardiology patients’ records and discharge summaries and evaluating whether targeted educational interventions could improve documentation rates. Records from September to December 2025 were retrospectively reviewed, including only patients who reported driving at the time of admission. Following the audit, an educational intervention and a discharge flowchart poster with a QR code linking to the latest DVLA guidance were introduced. The data audit cycle was repeated three times after the initial review, with ongoing educational interventions.

Results

Initially, 19/33 (57.6%) discharge summaries and 15/33 (45.5%) patient notes included driving restriction advice. After educational interventions, this increased to 15/21 (71.4%) discharge summaries and 11/21 (52.4%) patient notes. Documentation declined after some junior doctors changed, with the second cycle at 14/23 (60.9%) for discharge summaries and 13/23 (56.5%) for patient notes. A repeat of educational interventions led to marked improvement, reaching 24/27 (88.9%) for discharge summaries and 19/27 (74.4%) for patient notes.

Conclusions

Educational interventions are valuable, but regular reminders should be included, such as discussing driving advice during departmental orientation for new junior doctors. Patients can be engaged by placing posters that prompt them to ask doctors about driving guidance. Additionally, a QIP is recommended to assess the accuracy of driving advice compared with DVLA guidelines.

## Introduction

Around 7.6 million people in the UK are living with heart and circulatory diseases, according to the British Heart Foundation (BHF) data [1]. These heart and circulatory conditions, including electrophysiology disorders, cardiac ischemia, and heart failure, are characterized by a risk of sudden, disabling symptoms [2]. This has resulted in cardiovascular conditions, particularly syncope and arrhythmia, being associated with an increased incidence of car accidents [3]. For this reason, being diagnosed with a primary cardiac condition can affect a patient’s eligibility to drive according to the Driver and Vehicle Licensing Agency (DVLA) rules.

In the UK, traveling by car is the most common mode of transport, with 58% of all journeys made by car [4]. This means a significant proportion of patients diagnosed with cardiovascular conditions or undergoing cardiology interventions are vehicle drivers. Therefore, a lack of appropriate driving advice for patients with cardiovascular conditions may have serious patient and public safety as well as medicolegal implications.

While patients are legally responsible for notifying the DVLA of any condition that affects their fitness to drive, it remains the doctor’s duty to educate and inform patients of these limitations [5]. It is compulsory for all patients diagnosed with a cardiac condition to be informed of the driving restrictions, with the time frame of driving prohibition varying depending on the specific condition [5]. While the DVLA does not make it compulsory for driving advice to be documented, standard good clinical practice mandates that any important discussion, procedures, or investigations conducted should be clearly documented in the patient's record and discharge summary.

Despite this guidance, clinical practice experience and existing data suggest that provision of advice is often informal, not clearly documented, or omitted altogether, either due to oversight or doctors lacking knowledge of driving restrictions due to cardiovascular conditions. A review of studies on driving advice for cardiology patients identified one notable publication. Vusirikala et al., at Southmead Hospital, Bristol, highlighted the seriousness of this problem, as only 10% (9/92) of discharge summaries showed documentation of driving advice [6].

Based on that, a short QIP to assess whether documentation in both patient notes and discharge summaries serves as evidence that driving advice has been given to patients admitted under the cardiology service at a district hospital was conducted. This quality improvement project (QIP) aimed to assess the documentation of driving advice in cardiology patient records and discharge summaries and evaluate whether targeted educational interventions could improve documentation rates.

## Materials and methods

Study design and setting

This single-center retrospective QIP project at Altnagelvin Hospital, Northern Ireland, used the Plan-Do-Study-Act (PDSA) model to evaluate documentation of driving advice for cardiology patients in patients’ notes and discharge records. This project was conducted as a quality improvement initiative and therefore did not require formal ethical approval from the institution.

Study period

The complete QIP was carried out from September to December 2025. The baseline audit took place from September 6 to 9, 2025, followed by the first cycle audit between October 2 and 4, 2025. The second cycle audit occurred from November 16 to 20, 2025, and the third and final cycle audit was conducted from December 17 to 20, 2025.

Inclusion and exclusion criteria

During the QIP period, all adult male and female patients admitted to the cardiology unit were initially screened for eligibility. Inclusion criteria required documentation in patient records indicating that the individual was an active vehicle driver at the time of admission. Patients were excluded if records indicated they were not active vehicle drivers or if their driving status was undocumented.

Data audit

A nonprobability convenience sampling method was used due to its cost-effectiveness and ease of use in a short QIP. Double data extraction was performed by the author and a second independent reviewer. The collected data were compared, and any discrepancies were discussed to reach a consensus. A total of four data audit cycles were performed: a baseline audit and three subsequent cycles, with the fourth cycle being the final data audit due to time constraints. Each audit cycle manually reviewed patient electronic records from the prior two weeks. The process commenced with the identification of patients from archived ward files. Patient Health and Care numbers were utilized to locate and access electronic medical records. An initial screening determined whether individuals fulfilled the inclusion criteria; qualifying records were subsequently reviewed to ascertain whether driving advice was documented within the patient notes or discharge summary. The extracted data included patient demographics, cardiac conditions, and documentation of driving advice in patient notes and discharge summaries. All data were anonymized and systematically entered into Excel (Microsoft Corporation, Redmond, WA, USA) for subsequent analysis and graphical representation. Descriptive analysis of data was performed, with results presented in the form of a bar graph for patient demographics and cardiac conditions and a line graph for documentation of driving recommendations in patient records and discharge summaries.

Interventions for improvement

After the initial baseline data audit, interventions targeting junior doctors were implemented to improve the rate of documentation of driving advice in patient notes and discharge summaries. The interventions were repeated after the second data audit cycle to accommodate new junior doctors who had joined the department after the early November clinical rotation changeover.

**Figure 1:**
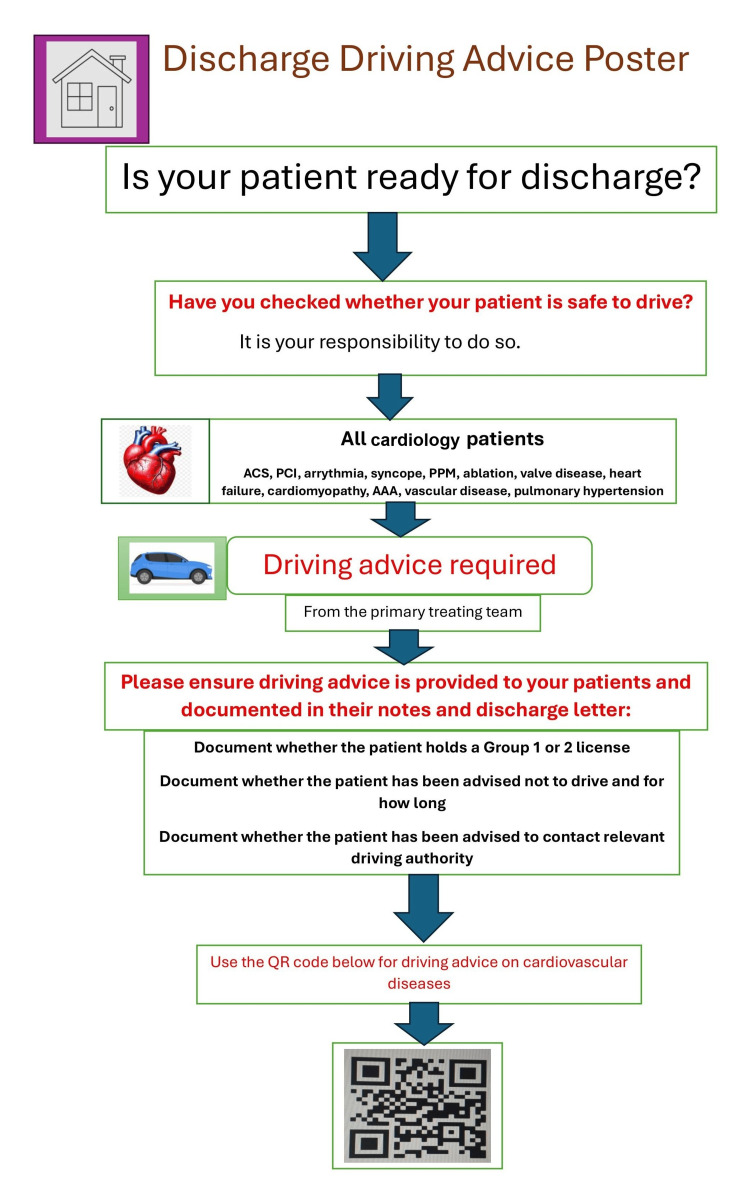
Reminder poster to give and document discharge advice AAA, abdominal aortic aneurysm; ACS, acute coronary syndrome; HTN, hypertension; PCI, percutaneous coronary intervention; PPM, permanent pacemaker

**Figure 2:**
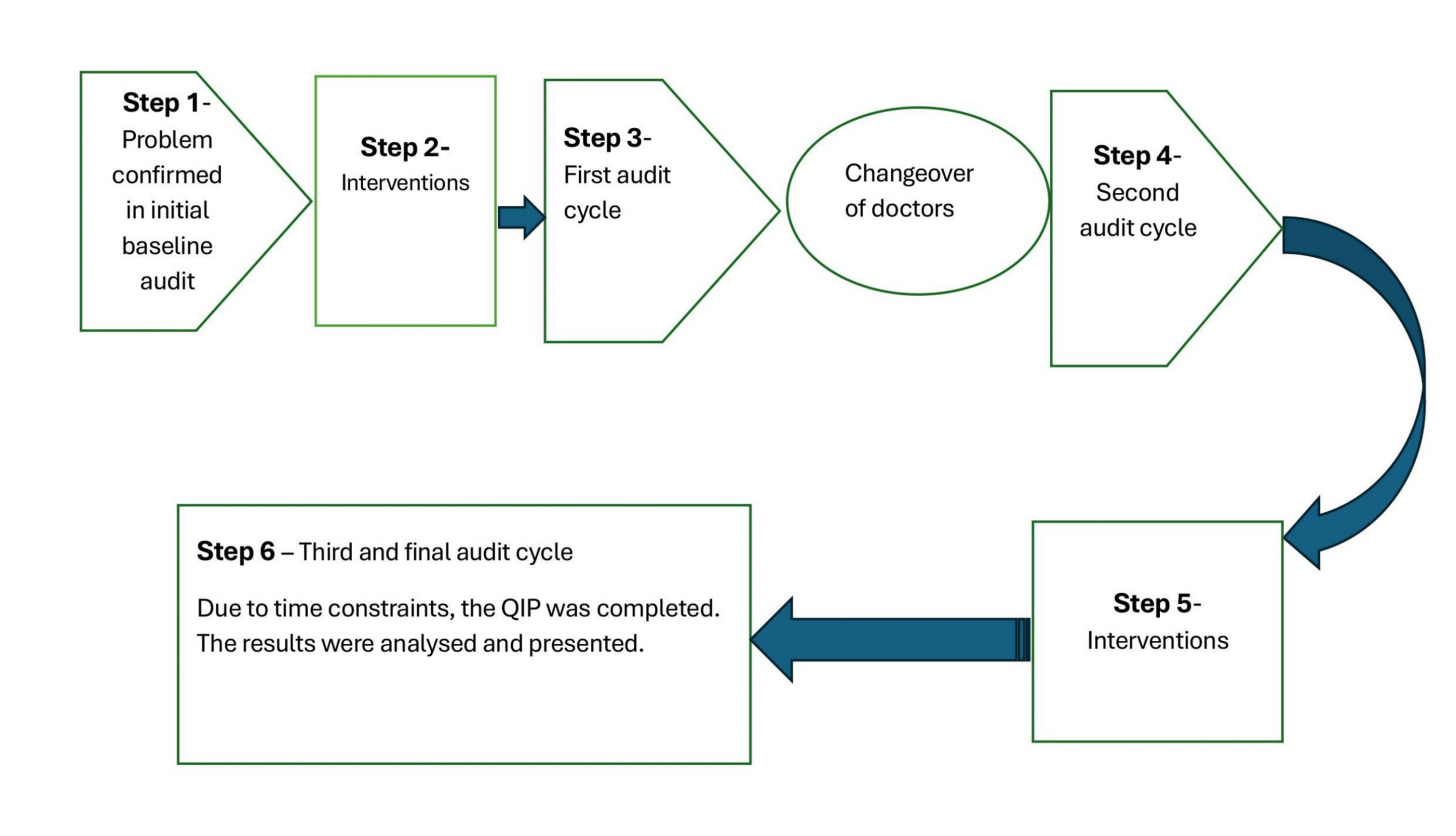
Schematic flowchart of the QIP showing the steps taken during the QIP project QIP, quality improvement project

The interventions featured a discharge driving advice poster with a QR code linking to up-to-date driving guidelines (Figure 1). This served as a prompt to provide and record driving advice in both patient notes and discharge summaries. Posters were placed near junior doctors’ workspaces and ward notice boards and were also included in an awareness email to junior doctors as part of the educational intervention efforts. Presentations were given at the monthly departmental teaching sessions in September and November 2025. This educational intervention was modelled after a previous study at Imperial College Healthcare NHS Trust, where similar strategies increased the rate of discharge advice from 3% to 79% [7]. Figure 2 shows a pictorial summary of the QIP schematic process.

## Results

The complete QIP comprised 104 patients. Acute coronary syndrome was the most prevalent diagnosis, affecting 58 patients (55.7%), with 33 males (56.9%) and 25 females (43.1%). Heart valve disease ranked second, observed in 18 patients (17.8%): 10 males (55.6%) and eight females (44.4%). Arrhythmia and syncope accounted for 15 cases (14.0%), with nine males (60%) and six females (40%). Heart failure was the least encountered cardiac condition, found in 13 patients (12.5%), with six males (46.2%) and seven females (53.8%).

**Figure 3:**
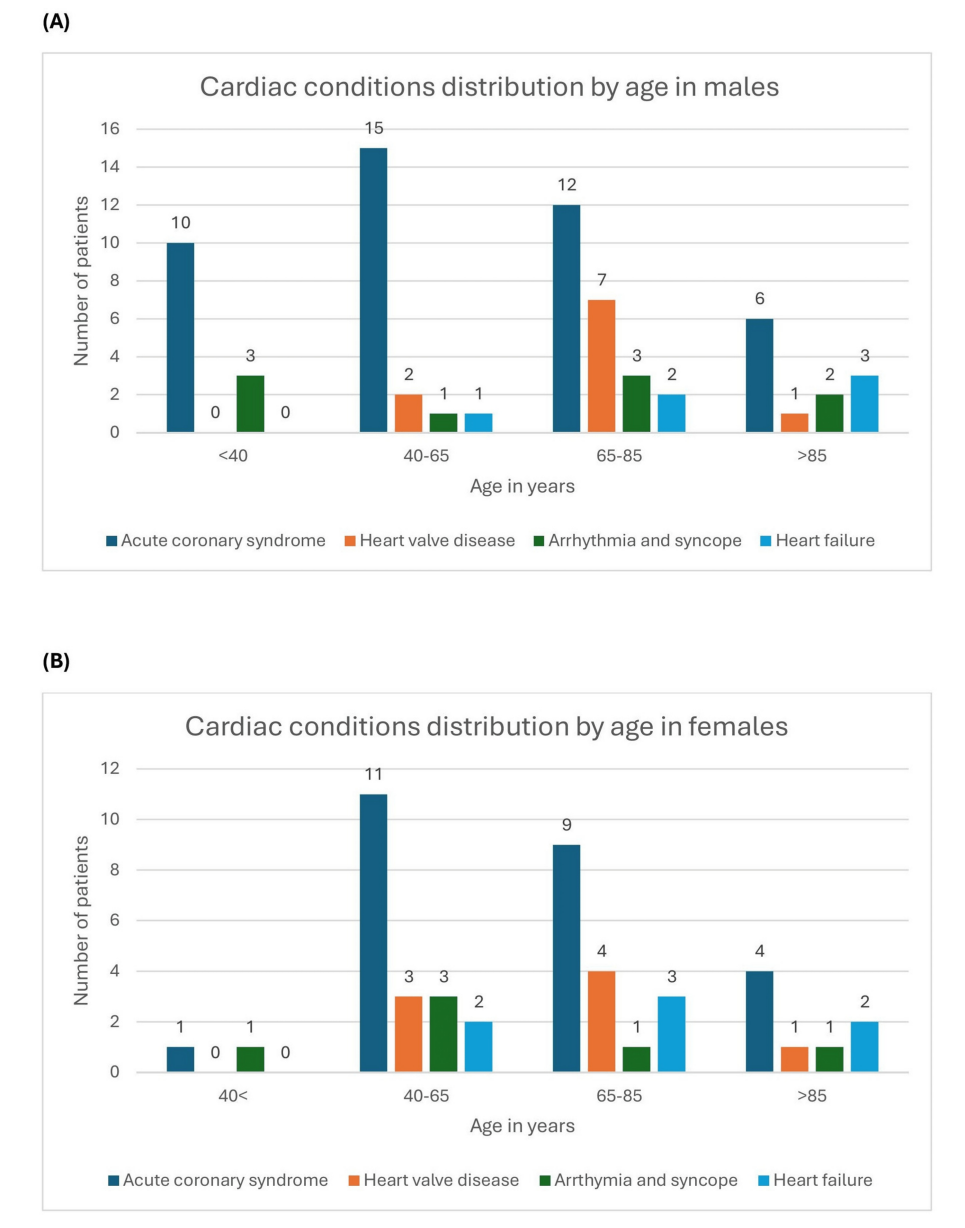
Distribution of cardiac conditions by sex and age range of patients reviewed during the QIP project QIP, quality improvement project

Regarding age distribution, the majority of cardiac conditions were noted in the 40-85 years age range, representing 79 individuals (76%). Patients under 40 years constituted the smallest group, with 15 records reviewed (14.2%). Within this younger cohort, arrhythmia and syncope were the most frequent cardiac diagnoses, comprising 11 cases (73.3%). Overall, males tended to be affected by cardiac conditions at a comparatively younger age than females (Figure 3).

**Figure 4:**
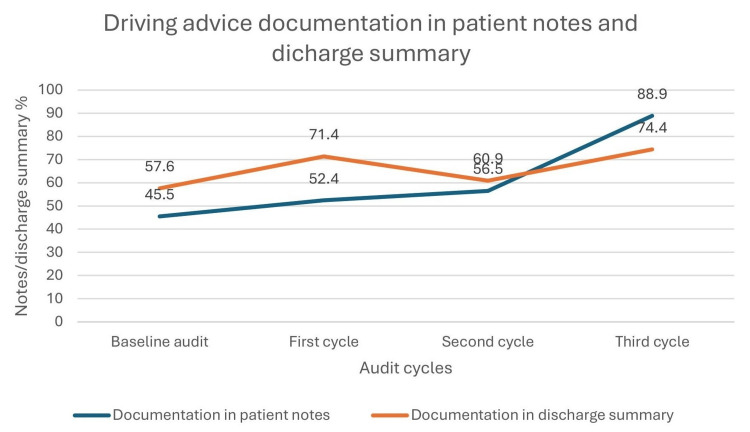
Documentation of driving recommendations in patients’ records and discharge summaries

Figure 4 shows driving advice documentation trends in patient notes and discharge summaries. Initially, at baseline, 19/33 (57.6%) discharge summaries and 15/33 (45.5%) patient notes included driving restriction advice. After educational interventions, this increased to 15/21 (71.4%) discharge summaries and 11/21 (52.4%) patient notes. Documentation declined after some junior doctors changed departments as part of their rotational training, with the second cycle findings standing at 14/23 (60.9%) for discharge summaries and 13/23 (56.5%) for patient notes. A repeat of educational interventions led to marked improvement, reaching 24/27 (88.9%) for discharge summaries and 19/27 (74.4%) for patient notes.

## Discussion

The QIP aimed to determine whether driving advice was provided to patients with cardiac conditions upon hospital discharge, as indicated by documentation in patient notes and discharge summaries. Among the reviewed records, acute coronary syndrome was the most common diagnosis, followed by heart valve disease, arrhythmia and syncope, and heart failure being the least frequently encountered cardiac condition, with more males being affected as compared to females. Regarding age distribution, the majority of cardiac conditions were observed in the 40- to 85-year age range. Patients under 40 years constituted the smallest group. Within this younger cohort, arrhythmia and syncope were the most frequent cardiac diagnoses. Overall, males tended to be affected by cardiac conditions at a comparatively younger age than females. These findings align with data published by the BHF in January 2026, which indicate that cardiac conditions are generally more prevalent among males [8]. Coronary artery disease, a precursor to acute coronary events, is the most common condition, affecting approximately one in eight males and one in fifteen females [8].

Baseline audits showed that the rate of driving advice recording in patient notes or discharge summaries was low, reflecting similar findings by Vusirikala et al. at Southmead Hospital, Bristol [6]. This issue of poor documentation of driving advice extends beyond cardiology and appears to be widespread. Research by Tahsin et al. at UHL Trust found that only 21 out of 42 patients undergoing inguinal hernia repair received driving advice at discharge [9]. Educational interventions and reminder tools, including a driving advice reminder poster with a QR code linking to guidelines and reminder emails, led to significant improvements in documentation during the first audit cycle. These strategies mirrored those used at Imperial College Healthcare NHS Trust, where discharge advice rates increased from 3% to 79% following the introduction of strategies, including an educational intervention poster [7].

However, in the second audit cycle, documentation rates unexpectedly declined in both patient notes and discharge summaries. Further investigation showed this drop was due to a changeover of junior doctors, and the new cohort of junior doctors had not participated in the previous interventions for change. In support of the observation of failure to sustain positive change after staff changeover, previous published research shows that staff turnover often disrupts the sustainability of positive project changes. Lennox et al., in their paper titled “Unpacking the ‘process of sustaining’-identifying threats to sustainability and the strategies used to address them: a longitudinal multiple case study,” found that when nurses and junior doctors left, continuity suffered, making it harder to communicate and implement initiatives consistently due to loss of experience and expertise, thus undermining initiative memory and leading to loss of positive improvements gained [10]. The educational interventions were repeated with the new cohort, followed by a third audit cycle, which again demonstrated improved documentation of driving advice. No further audits were performed because of time constraints.

Limitations

The study’s small sample size from a single ward and lack of statistical analysis limits the generalizability of results, and the findings should be interpreted cautiously. There was also a possibility of observer bias affecting the results. In addition, patients transferred elsewhere to other wards were excluded, though they represent an important group whose discharge teams may overlook driving advice. Furthermore, relying on documentation in patient notes and discharge summaries as evidence that driving advice had been given meant that patients who received verbal instructions without documentation were considered as not having received any advice. Lastly, the QIP educational interventions included only junior doctors, excluding other staff such as physician assistants who frequently complete discharge summaries, as well as nurses and physiotherapists who are involved in patient discharge from the hospital.

Recommendations

Involving nursing staff, physiotherapists, and occupational therapists in educational interventions may improve audit outcomes by helping remind doctors to provide driving advice. Patient-focused posters can prompt individuals to ask their doctors about driving guidance. A follow-up QIP could assess whether discharge advice aligns with DVLA standards by contacting discharged patients to verify the actual advice they were given.

## Conclusions

This QIP identified significant challenges in providing driving advice to patients with cardiovascular conditions at the point of hospital discharge, highlighting potential risks to both patient and public safety, as well as medicolegal concerns. The project also demonstrated that implementing straightforward educational interventions and reminder tools can markedly enhance the documentation of driving advice for cardiology patients. Nevertheless, ensuring long-term sustainability necessitates embedding these practices within routine clinical workflows, such as through electronic prompts and structured discharge templates. Because these results are based on just one center, additional studies are suggested to assess how common and significant insufficient driving advice is for cardiovascular patients after leaving the hospital.
